# A Case of Low-Grade Appendiceal Mucinous Neoplasm That Led to Surgery After 12 Years of No Treatment

**DOI:** 10.7759/cureus.43024

**Published:** 2023-08-06

**Authors:** Akihiro Makino, Takuya Okumura, Kimihiro Yamashita, Jun Isogaki, Akihiro Kawabe

**Affiliations:** 1 Surgery, Fujinomiya City Hospital, Shizuoka, JPN

**Keywords:** laparoscopic surgery, ileocecal department resection, mucinous adenocarcinoma of appendix, peritoneal pseudomyxoma, low-grade appendiceal mucinous neoplasms

## Abstract

Low-grade appendiceal mucinous neoplasm (LAMN) is a relatively rare, non-invasive appendiceal tumor. We experienced a case of LAMN that led to surgery after 12 years of no treatment. Until now, LAMN has been reported to progress more slowly than other tumors, but there have been no reports of long-term follow-up of appendiceal tumors without treatment. Although the tumor had grown over the course of 12 years, there was no mixing or migration of other histological types, and it did not lead to pseudomyxoma peritonei. As this course is considered to be relatively rare, we report it along with a literature review.

## Introduction

Appendiceal mucinous neoplasm is a condition in which mucus accumulates in the appendix and the appendix is dilated. It is found in 0.2-0.3% of appendectomy specimens and is a relatively rare disease [1]. In the WHO classification and the colorectal cancer treatment regulations eighth edition or later, appendiceal mucinous neoplasm is classified into mucinous adenocarcinoma (MACA) with obvious atypia and other low-grade appendiceal mucinous neoplasm (LAMN) [2]. LAMN is a concept adopted from the eighth edition of the Japanese Classification of Colorectal Carcinoma in 2013, and in consideration of consistency with the WHO classification, the previous names mucocystadenoma and mucocystadenocarcinoma were eliminated. Most of the mucocystadenoma and some mucocystadenocarcinoma were considered to be included in LAMN [3]. LAMN is nearly synonymous with G1 (well-differentiated) appendiceal mucinous carcinoma in malignant epithelial tumors [4].

We treated a case of LAMN requiring surgery after 12 years of no treatment. As this course is relatively rare, we report it along with a literature review.

## Case presentation

A 62-year-old male with a medical history of bilateral inguinal hernia surgery (inguinal incision) had right lower abdominal pain from the morning of the previous day and was referred to our hospital for suspected appendicitis. He visited our hospital 12 years ago for abdominal pain, and at that time, a detailed examination by lower digestive endoscopy showed no abnormalities. On the current presentation to the hospital, tenderness with rebound pain was observed in the right lower abdomen. Blood tests showed a white blood cell count of 14,600/μL, C-reactive protein of 13.15 mg/dL, carcinoembryonic antigen of 3.3 ng/mL, and cancer antigen of 19-9 13.1 U/mL, and there were no other major outliers.

**Figure 1:**
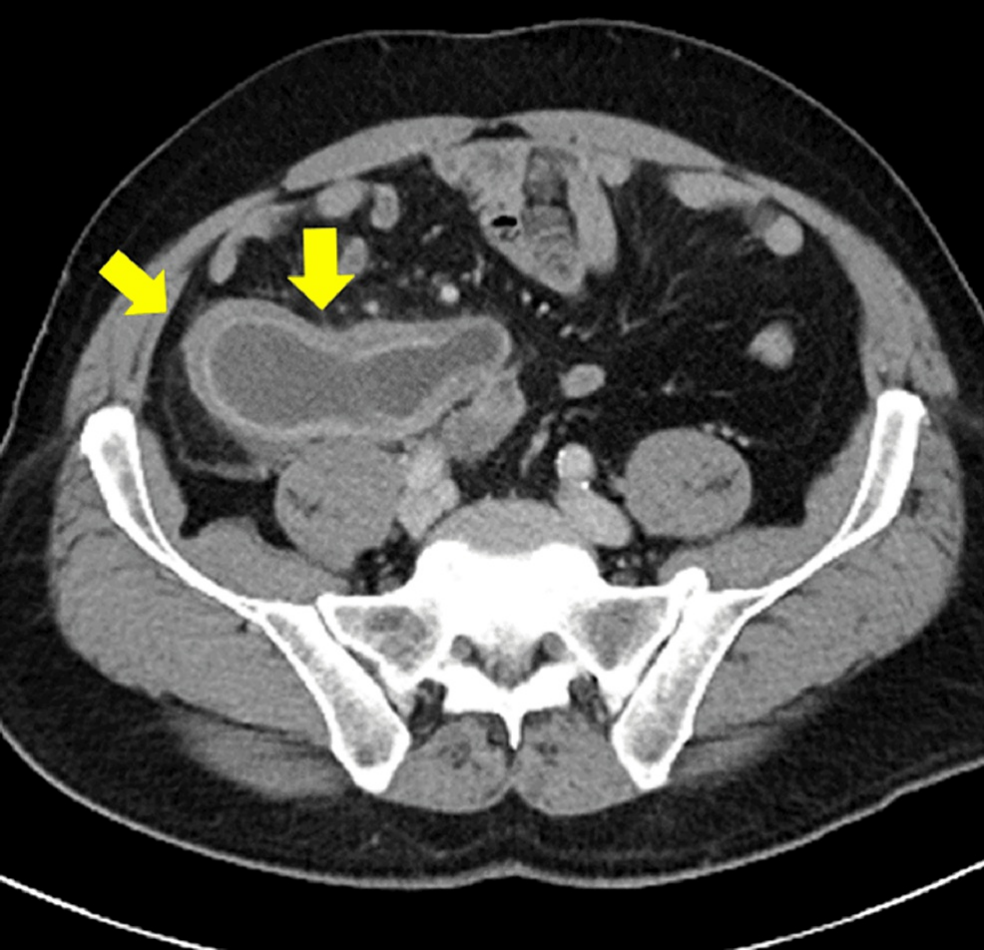
Contrast-enhanced computed tomography at the present time (axial image).

**Figure 2:**
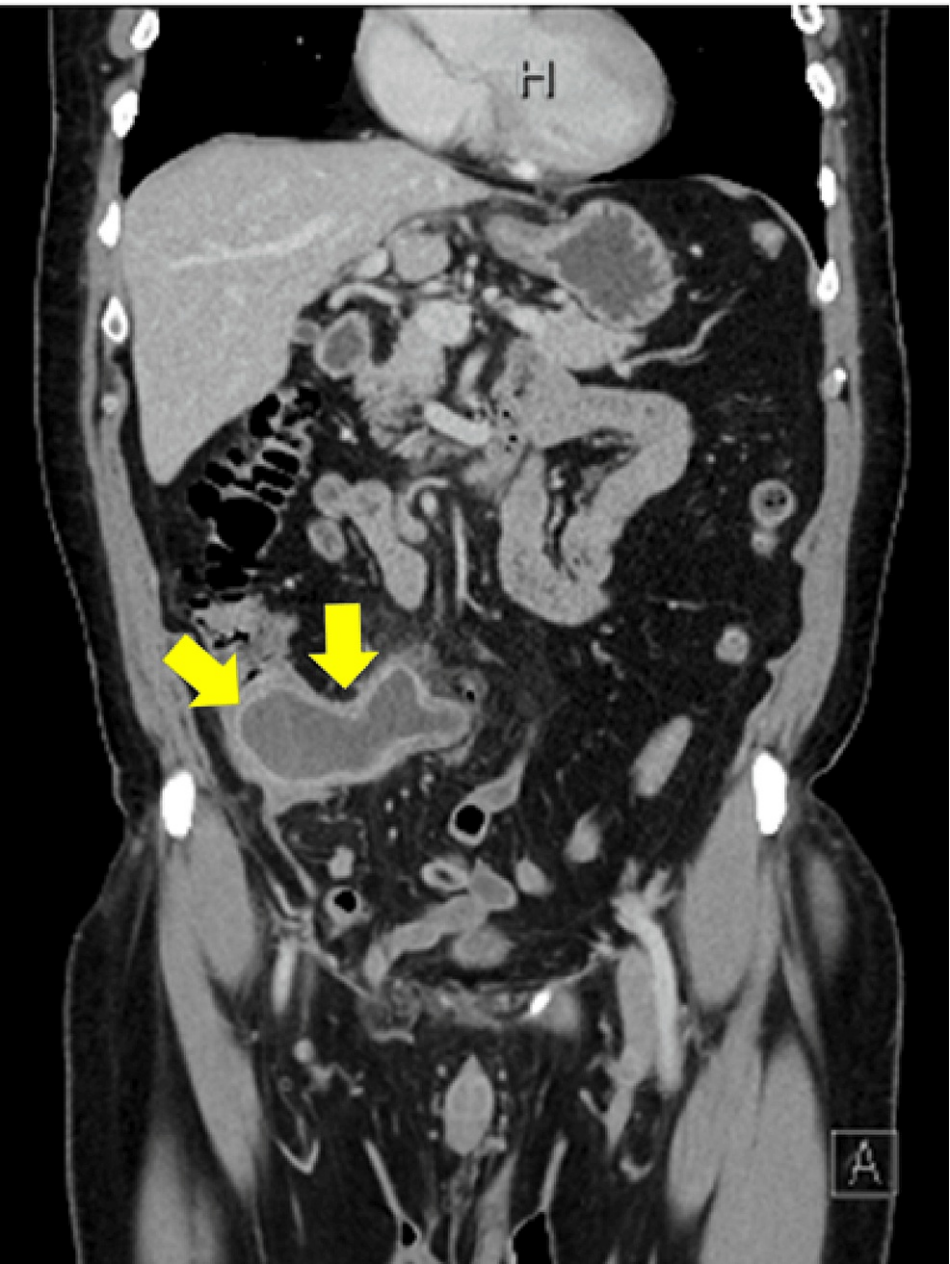
Contrast-enhanced computed tomography at the present time (coronal image).

**Figure 3:**
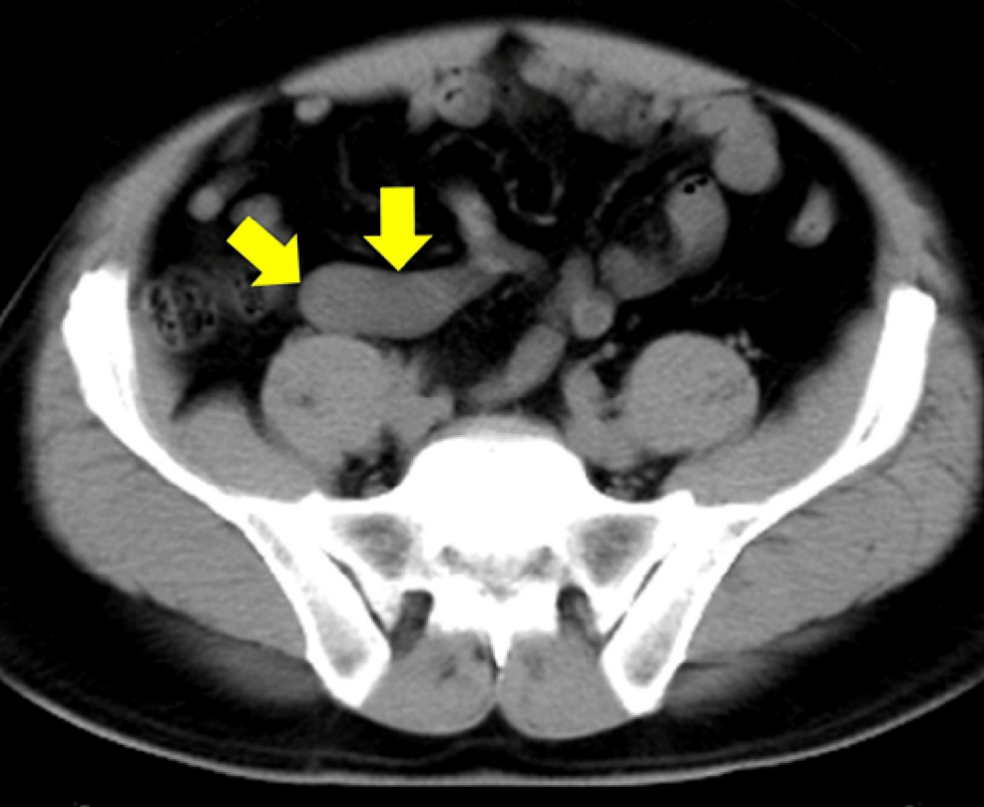
Plain computed tomography 12 years ago (axial image). A plain CT scan obtained 12 years ago showed that the appendix had enlarged to 20 mm (>13 mm) in size, and although wall calcification was not observed, the increase in the concentration of surrounding adipose tissue was not noticeable. These findings suggested appendiceal mucinous neoplasm.

Contrast-enhanced computed tomography (CT) revealed that the appendix was enlarged to a maximum diameter of 45 mm and the wall was thickened. The appendix lumen had expanded to 32 mm, and homogeneous fluid was stored inside. There were no fecal stones, and the increase in the concentration of the surrounding adipose was not very noticeable. No obvious enlarged lymph nodes were observed in the ileocolic artery area, and ascites were not observed (Figures 1, 2). We suspected imminent rupture of an appendiceal mucinous neoplasm. Retrospectively, a plain CT scan at the time of the visit 12 years ago showed that the appendix had enlarged to 20 mm (>13 mm) in size, and although wall calcification was not observed, the increase in the concentration of surrounding adipose tissue was not noticeable, suggesting appendiceal mucinous neoplasm (Figure 3). At that time, the appendiceal mucinous neoplasm had not been diagnosed, and the tumor had increased in the course of 12 years.

**Figure 4:**
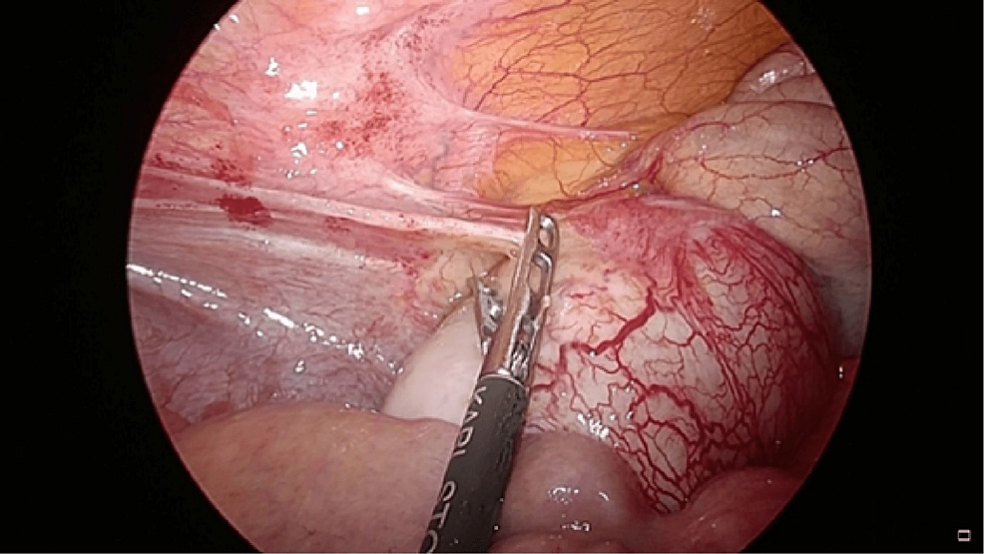
Intraoperative image. Observation of the abdominal cavity revealed a swollen appendix. We dissected the ileocecal laparoscopically and transferred to extracorporeal operation.

**Figure 5:**
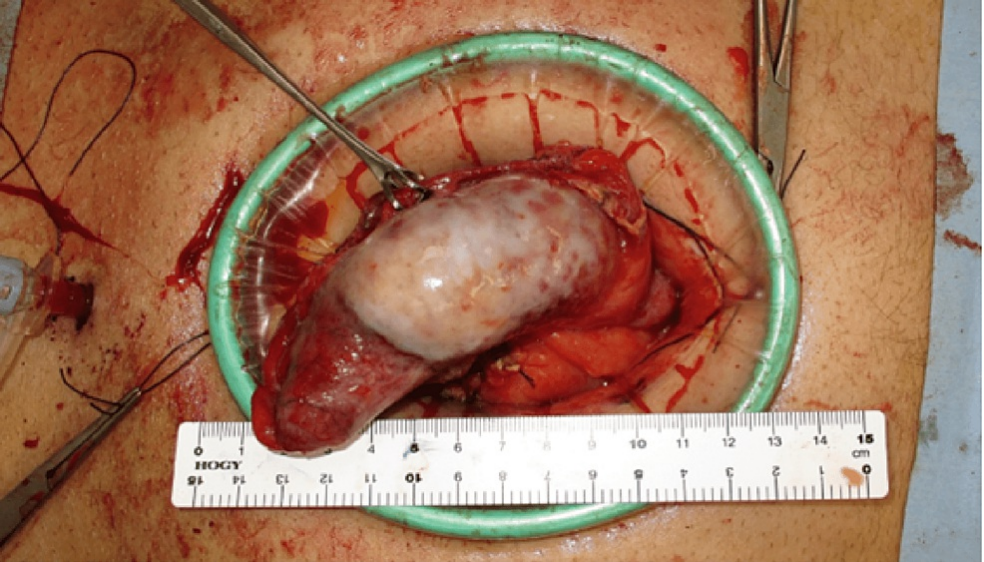
Intraoperative image (cranial side is on the right). Ileocecal resection and anastomosis were performed under direct vision, and lymphadenectomy was done at about the D1 levels.

**Figure 6:**
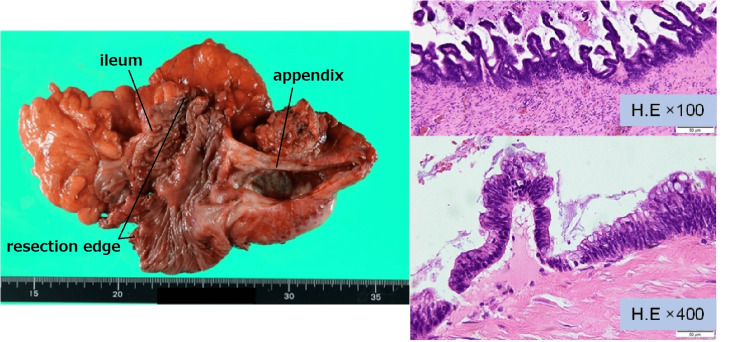
Postoperative pathological image. The appendix of the resected specimen contained mucus. The tumor grew with a corrugated structure, and showed an expansive growth pattern, with no invasive growth. A partly mucoid reticulum was also observed. Tumor cells were pseudostratified and had short spindle-shaped nuclei. Tumor cell atypia was weak and the histopathological diagnosis was low-grade appendiceal mucinous neoplasm. The resection was negative, and no lymph node metastasis was observed.

There was an option to perform surgery after conservative treatment, but it was clear that the appendiceal mucinous neoplasm was growing. As the risk of rupture was considered, we decided to perform emergency surgery. After laparoscopic dissection of the cecum, ileocecal resection and anastomosis were performed under direct vision, and lymphadenectomy was done at about the D1 levels (Figures 4, 5). The appendix of the resected specimen contained mucus, and the histopathological diagnosis was LAMN (Figure 6).

The resection edge was negative, and no metastasis was observed in the regional lymph nodes. After surgery, paralytic ileus (Clavien-Dindo Grade II) was observed, but the patient was discharged on postoperative day 21. Twelve months have passed since the operation, and the patient is alive without recurrence.

## Discussion

Although LAMN is considered to be slow-growing compared to other tumors [4,5], according to previous reports, the course until the development of LAMN at the appendectomy stump [5] and the occurrence of pseudomyxoma peritonei (PMP) after focal resection was long [4]. There have been no reports of long-term follow-up of appendiceal tumors without treatment. In this case, which had a long treatment-free period of 12 years, there are two interesting points. One is that there was no mixing or migration of other histological types, and the other is that it did not lead to PMP.

An appendiceal tumor was clearly suspected from the preoperative stage in this case. As it was difficult to distinguish benign from malignancy, resection with a margin, and resection of ileocecal was required because of proximity to the Bauhin valve. A protective operation was required to prevent myxoma rupture. Ileocecal resection with D1 lymphadenectomy was performed under direct vision with small laparotomy, and there was concern that the lymph node dissection area was insufficient. In principle, surgical resection is the treatment for LAMN, but there are no clear standards for the method of resection [6,7]. If MACA is diagnosed on postoperative histopathological examination, additional resection including lymph node dissection is indispensable [8]. However, there is no evidence regarding additional treatment when LAMN is diagnosed, and it is difficult to determine whether additional resection including lymph node dissection is reasonable or not [8]. Past reports have shown that lymph node dissection was performed on LAMN, and no lymph node metastasis was pointed out, suggesting that the need for lymph node dissection may not be high [6,7]. Additional lymph node dissection is considered unnecessary for accidentally discovered LAMN after appendectomy. As a case of consideration for additional surgery, a positive appendectomy stump may be considered, but there are also reports that this does not pose a risk of occurrence of PMP [9,10], and it is necessary to reconsider. According to a previous study, as it is difficult to predict which patients will progress to PMP, cross-sectional imaging monitoring is recommended for all patients [9].

Although there is no clear evidence at present whether additional ileocecectomy is necessary at a later date if appendectomy is performed as appendiceal mucinous neoplasm and pathologic findings indicate appendiceal cancer, there is a report that it responds according to the following criteria [4]: (1) if LAMN is diagnosed, stump negative, and there are no lymph nodes suspected of metastasis on CT, the rate of lymph node metastasis of LAMN is extremely low, making additional resection not necessary. However, if the myxoma is perforated intraoperatively or mucus is sprayed, there is a possibility of seeding recurrence, making strict image follow-up crucial. (2) In the case of appendiceal cancer other than LAMN, if the depth is greater than the submucosal layer, there is a risk of lymph node metastasis, so additional resection is considered as with other colorectal cancers. (3) If obvious dissemination is observed at the time of the first resection, it is not necessary to stick to lymph node dissection, but appendectomy or ileocecectomy, which is the primary lesion, is performed as much as possible. This is because by determining the histological type and grade by the pathological diagnosis of the primary lesion, it is possible to predict the prognosis and determine the treatment strategy such as chemotherapy. Hence, it was judged that additional surgery including lymph node dissection was not required in our case.

## Conclusions

This is the first report of an appendiceal tumor with a long treatment-free period of 12 years. The pathogenesis of LAMN remains largely unknown, and although there have been reports suggesting a transition to other histological types in the past, the details are still unknown. In this case, there was no mixing or migration of other histological types, and it did not lead to PMP.
